# Supplement use in relation to dietary intake in pregnancy: an analysis of the Swedish GraviD cohort

**DOI:** 10.1017/S0007114523001794

**Published:** 2024-01-28

**Authors:** Mathilda Forsby, Anna Winkvist, Linnea Bärebring, Hanna Augustin

**Affiliations:** Institute of Medicine, University of Gothenburg, Gothenburg 40530, Sweden

**Keywords:** Nutrients, Diet, Dietary supplements, Maternal health, Pregnant women

## Abstract

We aimed to study supplement use in relation to dietary intake among pregnant women in Sweden, and adherence to the Nordic Nutrition Recommendations among supplement and non-supplement users. Pregnant women were recruited at registration to antenatal care in 2013–2014. In third trimester, supplement use was collected using a questionnaire, and dietary intake was collected using a FFQ. The majority (64 %) of the 1044 women reported use of one or more supplements. Among all, 0–23 % reported dietary intakes above recommended intake (RI) of vitamin D, folate, Fe and Se. Median dietary intakes of thiamine (1·4 *v*. 1·3 mg *P* = 0·013), phosphorus (1482 *v*. 1440 mg *P* = 0·007), folate (327 *v*. 316 µg *P* = 0·02), Fe (12 *v*. 11·5 mg *P* = 0·009), Mg (361 *v*. 346 mg *P* < 0·001) and Zn (10·7 *v*. 10·4 mg *P* = 0·01) were higher among supplement users compared with non-users. Larger proportions of supplement users than non-users adhered to RI of dietary intakes of thiamine (42 % *v*. 35 % *P* = 0·04) and Mg (75 % *v*. 69 % *P* = 0·05). Among non-users, a minority had dietary intakes above RI for vitamin D (6 %), folate (10 %) and Fe (21 %). The majority (75–100 %) of supplement users had total intakes above RI for most nutrients. In conclusion, supplement use contributed substantially to reaching RI for vitamin D, folate and Fe. Supplement users had a higher dietary intake of several nutrients than non-users. This highlights that non-supplement users are at risk of inadequate nutrient intakes during pregnancy, suggesting a need for heightened awareness of nutritional adequacy for pregnant women.

## Graphical abstract

**Figure:**
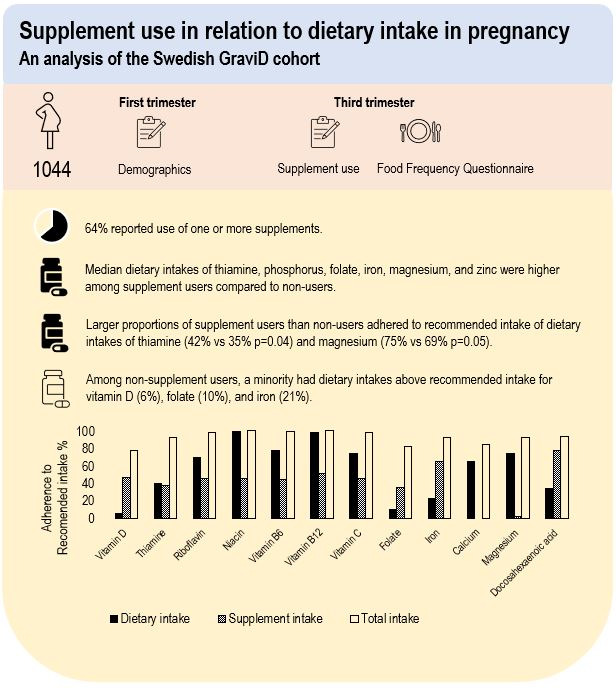

Dietary supplements are concentrated forms of vitamins and minerals and aim to complement nutrient intake from diet^(1)^. Nutrient intake from diet is sufficient for the majority of people if dietary recommendations are followed^(2)^. However, exceptions exist where dietary supplements are needed to ensure adequate nutritional intake. During pregnancy, the requirements of certain nutrients are increased to meet maternal demands while supporting development of the fetus^(3)^. Important key nutrients during pregnancy include folate to prevent neural tube defects^(4)^, iodine and DHA for fetal brain development^(5,6)^, vitamin D and Ca for fetal and maternal bone mineralisation^(7,8)^ and Fe for reduced risk of maternal anaemia^(9)^.

Insufficient nutritional intakes and deficiencies among pregnant women in low- and middle-income countries are highly recognised as public health problems^(10)^. However, a systematic review and meta-analysis indicated that pregnant women from developed countries are at risk of inadequate micronutrient intakes^(11)^. More specifically, reported intake of vitamin D, folate and Fe was found to be lower than the nutritional recommendations in most countries included. In Sweden, pregnant women are recommended a daily intake of 400 μg folic acid preconceptionally until gestational week 12^(12)^. Also, iodine-fortified salt is recommended for all and supplementation of vitamin D and DHA if intakes from food sources are insufficient^(12)^. Screening for Fe deficiency is performed throughout pregnancy by the Swedish antenatal care and supplements are prescribed when needed^(12)^. Supplements are purchased by the pregnant woman herself.

Previous studies indicate that use of dietary supplements during pregnancy in Nordic countries is common, ranging from 78 % to 95 % of pregnant women^(13–17)^. Results from the Norwegian Mother, Father and Child Cohort Study showed that dietary supplements had a substantial contribution to the total intake of vitamin D, folate, DHA, EPA, vitamin B6, copper and Fe among pregnant women^(14)^. Despite the increase in total nutrient intakes from dietary supplements, more than 25 % of the supplement users still did not meet the recommended intakes (RI) for vitamin D, folate and iodine^(14)^.

Results from our group^(16)^ and others^(13,15,18)^ have previously shown associations between use of dietary supplements during pregnancy and certain socio-demographic factors. For instance, supplement use has been related to parity^(13,16,18)^, birthplace^(16)^, education^(13,15,16,18)^, employment^(16)^, age^(13,15,18)^, BMI^(13,15)^ and smoking during pregnancy^(13)^.

Routine visits at the Swedish antenatal care include health-promoting consultation of dietary habits and supplementation, and assessment of nutritional status^(19)^. However, the choice of dietary intake and supplement use are largely autonomous practices, which requires the pregnant woman herself to implement a total intake in line with recommendations^(20)^. The third trimester of pregnancy is an important period for assessing nutritional intake, as it corresponds to a period of rapid growth and development of maternal and fetal tissues^(21)^. Furthermore, most of the fetal Ca transfer takes place in third trimester^(22)^, while concurrently, the maternal Fe requirement reaches its peak, as the fetus accumulates Fe for its early life needs^(23)^. Previous Swedish studies on supplement use during pregnancy have mainly been restricted to frequency and prevalence of supplement use^(13,16,24)^, whereas the total nutrient intake from supplemental and dietary sources has rarely been reported^(25)^. The aims were therefore to study supplement use and adherence to the Nordic Nutrition Recommendations in third trimester of pregnancy and to study differences in dietary and total intakes of nutrients between non-supplement users and supplement users; the latter defined as ‘any type’ or ‘nutrient-specific’ supplement users.

## Method

### Study population and study design

Data were drawn from the population-based GraviD cohort where pregnant women in the south-west of Sweden were recruited during Autumn 2013 and Spring 2014. Recruitment and data collection were carried out at routine visits to the antenatal care. All women in gestational weeks ≤ 16 within the study area were eligible for inclusion. Verbal and written study information was given by midwives at registration for antenatal care at one of the participating units. Study information and consent forms were available in nine languages and interpreters were provided if required, according to antenatal care standard routines. Written consent was obtained from all study participants. The study was approved by the Regional Ethics committee in Gothenburg, Sweden (protocol 2019-05219, 879-11, T439-13, T085-14), and all procedures were conducted in accordance with the Declaration of Helsinki.

Data on socio-demographic factors were collected in first trimester using a questionnaire. Information of age, weight, height, parity and tobacco use in early pregnancy were obtained from medical records.

### Supplement use and assessment of dietary intake

In third trimester (gestational week > 31), the women answered an analogue questionnaire including supplement usage during the past 2 months. The women were asked if they used supplements and if so, to specify brands, doses and duration of use. Reported supplements that either singly or in combination contained vitamins, minerals, *n*-3 fatty acids or botanical substances were defined as supplements. The analyses of supplementation use included supplements with a single micronutrient or several combined (referred to as multivitamin-mineral supplement). Nutrient proportions derived from multivitamin-mineral supplements were determined based on the number of women using a specific nutrient from multivitamin-mineral supplements divided by the number of women who reported the same nutrient from both a single nutrient supplement and multivitamin-mineral supplements. *N*-3 fatty acids were only analysed as single supplement since those were not present in any multivitamin-mineral formulations (online Appendix Table 1). Information on nutrient content of reported supplements was obtained from the website of the provider or manufacturer. Estimations were made when a frequency but not an amount was specified or when supplements were recorded without a corresponding brand (online Appendix Table 2). When the amount was not recorded, the most frequently reported dose for each specific supplement was used. When brand was not recorded for multivitamin-mineral supplements, the most commonly reported prenatal multivitamin-mineral was used (online Appendix Table 3). Two definitions of supplement users were applied. The first definition, any supplement users, was defined as those reporting use of at least one (or more) supplement of any type. The second definition, nutrient-specific supplement users, was defined as those reporting use of a specific nutrient (e.g. vitamin D) either in a single nutrient supplement or as part of a multivitamin-mineral supplement. Non-supplement users were defined as those reporting no use of any type of supplement.

In third trimester, information on dietary intake was collected using a validated online semi-quantitative FFQ^(26,27)^. Information regarding the FFQ was provided at a routine visit to the antenatal care, but the questionnaire was to be answered after the visit and took approximately 20 min to complete. The FFQ was only available in Swedish, and women were encouraged to use help from a relative or friend if needed. The FFQ assessed the dietary intake during the past 2 months and included 174 food items and nine intake frequency categories. Portion sizes were estimated using either pictures or standard portion sizes, depending on the food item. Questions about the use of salt were included, but not whether the salt was iodised or not. Since iodised salt in Sweden is a large source for iodine intake^(28,29)^, intake of iodine was excluded for the current analyses. The FFQ used the National Food Agency’s food database to calculate intakes of energy, macro- and micronutrients^(30)^.

**Fig. 1.**
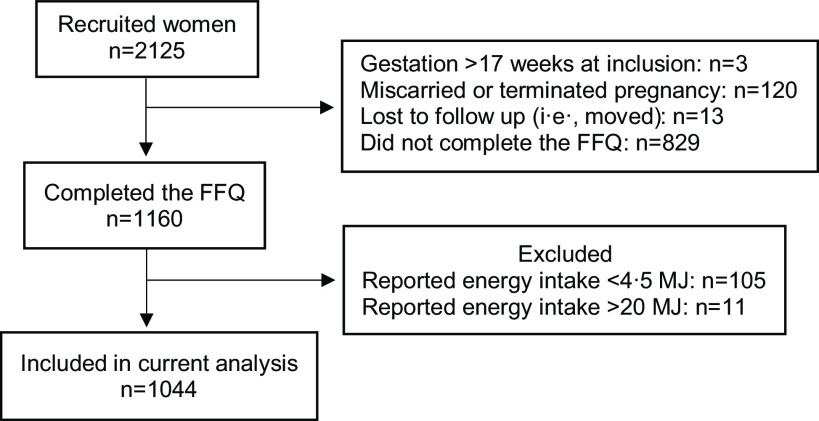
Flow chart showing inclusion of study participants in current analyses.

Inclusion criterium for the current analyses was a completed FFQ. Reported energy intake below 4·5 MJ (1070 kcal) (*n* 105) and over 20 MJ (4760 kcal) (*n* 11) was regarded as implausible intakes and was thus excluded^(17,31)^ (Fig. 1).

RI for pregnant women and average requirement (AR), lower intake level (LI) and upper intake level (UL) for women in reproductive age were derived from Nordic Nutrition Recommendations 2012^(32)^. In Nordic Nutrition Recommendations 2012, there is no RI for Fe concerning pregnancy; therefore, RI for women of reproductive age was used^(32)^.

### Statistical analysis

Data are presented as median and 25th and 75th percentiles since not all variables were normally distributed. Dietary intake was energy adjusted according to the residual method^(33)^. Differences in continuous and categorical variables capturing maternal characteristics between any supplement users and non-supplement users were tested by Mann Whitney U test and *χ* ^2^ test, respectively. Mann Whitney U test was also performed to compare estimated dietary intake between any supplement users and non-supplement users. Differences in adherence to RI and AR between any supplement users and non-supplement users were tested by *χ* ^2^ test.

To evaluate associations between dietary intake and nutrient-specific supplement use (e.g. Fe, Ca, Mg, folic acid, vitamin C, vitamin B complex, vitamin D and *n*-3 fatty acids), univariable and multivariable logistic regression analyses were used. The dependent variable was supplement use (nutrient-specific and non-supplement use) and continuous dietary intake as independent variable. The first model included dietary intakes adjusted for total energy intake and the final was also adjusted for potential confounders. Potential confounders were identified by a directed acyclic graph^(34)^ and included age and BMI in first trimester (gestational week ≤ 14), gestational weight gain (GWG), education, parity and origin. GWG was calculated as body weight in gestational week 37 ± 2 weeks minus weight in gestational week ≤ 14. Age, BMI and GWG were included as continuous variables. Categorical variables were education (university education or no university education), parity (nulliparous or parous) and origin defined by country of birth (born in Sweden or in another country). No university education, parous and born in Sweden were set as reference categories. Potential confounders were tested by the purposeful selection method for model-building, called the Bursac method^(35)^. All variables that were indicated as potential confounders by the directed acyclic graph were also identified by the Bursac method. Hence, age, BMI, GWG, education, parity and origin were included as confounders in the adjusted logistic regression models.

Power calculations were performed for the primary aim of the GraviD cohort and indicated that a sample size of 2000 had an 85 % power to detect a doubled incidence of pregnancy-induced hypertension among women with vitamin D deficiency^(36)^. The obtained sample size is believed to be sufficient for the current analyses^(16)^. Statistical analyses were performed using IBM SPSS Statistics version 28.0 (IBM Corp). No correction for multiple testing was made.

## Results

Of the 2125 pregnant women recruited in the GraviD cohort, 1044 were included in this analysis after exclusion of women with incomplete FFQ and implausible energy intakes (Fig. 1). At registration to the antenatal care in early pregnancy, median age (p25, p75) was 32 (29, 35) years and BMI 23·4 (21·3, 25·7) kg/m^2^ (Table 1). The majority (70 %) had a university education and few women (4 %) reported current use of tobacco. Less than half (43 %) were nulliparous.

**Table 1.** Participant characteristics among all women, any supplement users and non-supplement users (Median and percentiles; numbers and percentages)

	All women (*n* 1044)		any-SU (*n* 665)		non-SU (*n* 379)		Difference between any-SU and non-SU
	Median	p25, p75	Median	p25, p75	Median	p25, p75	*P* *
Age T1 (years)	32	29, 35	32	29, 35	31	28, 34	< 0·001
BMI T1 (kg/m^2^)	23·4	21·3, 25·7	23·2	21·2, 25·6	23·5	21·4, 25·9	0·23
Gestational weight gain (kg)†	13·0	11·0, 16·0	13·0	11·0, 16·0	13·0	11·0, 16·0	0·67
	%	*n*	%	*n*	%	*n*	*P* ‡
Origin							0·12
North Europe	86·0	898	86·0	572	86·0	326	
Continental Europe	5·3	55	6·2	41	3·7	14	
Asia	5·9	62	5·9	39	6·1	23	
America	1·5	16	1·1	7	2·4	9	
Africa	1·2	13	0·9	6	1·8	7	
Education level							0·01
Primary	1·4	15	0·8	5	2·6	10	
Secondary	28·4	296	26·8	178	31·2	118	
University	70·2	732	72·5	482	66·1	250	
Tobacco use T1	3·5	37	2·7	18	5·0	19	0·05
Nulliparity	42·9	448	44·7	297	39·8	151	0·13

any-SU, any supplement users; non-SU, non-supplement users; T1, trimester one.

* Assessed by Mann Whitney U test.

† Weight in gestational week 37 ± 2 weeks minus weight in gestational week ≤ 14.

‡ Assessed by *χ* ^2^ test.

In total, 64 % reported use of one or more type of supplements in third trimester. Among supplement users, 76 % used one, 21 % used two and 3 % used three to five supplements. The most frequently used supplements contained Fe (52 %), folic acid (50 %) and vitamin D (45 %) either as a single nutrient supplement or as part of a multivitamin-mineral supplement (Table 2). Use of a multivitamin-mineral supplement was overall reported by 40 % of the women. Large proportions of the nutrients obtained from supplementary sources (ranging between 87 % and 99 %) were derived from multivitamin-mineral supplements. The proportion derived from multivitamin-mineral supplements was somewhat lower for folic acid (77 %) and Fe (60 %) supplementary sources. Women categorised as supplement users were older and had a higher education than non-users (Table 1).

**Table 2.** Proportions of reported supplement use in third trimester as a single nutrient supplement and/or part of multivitamin-mineral supplement

Supplement use	Proportion % using the supplements as single nutrient supplement or part of multivitamin-mineral supplement	*n* users/*n* all
Any supplement (one or more)	64	665/1044
Vitamin D	45	469/1044
Folic acid	50	518/1044
*n*-3 fatty acids	5	53/1044
Vitamin B complex	42	434/1044
Vitamin C	40	420/1044
Mg	39	403/1044
Ca	38	400/1044
Fe	52	547/1044
Other (herbs, aloe vera, nettles, algae)	1	10/1044

Estimated dietary intakes during third trimester are presented in Table 3. Compared with non-users, supplement users had higher energy-adjusted median intakes of fibre, polyunsaturated fat, thiamine, phosphorus, folate, Fe, Mg and Zn.

**Table 3.** Estimated dietary intake adjusted for total energy intake among all women, by any supplement users and non-supplement users (Medians and percentiles)

	All women (*n* 1044)		any-SU (*n* 665)		non-SU (*n* 379)		Difference* between any-SU and non-SU
Dietary intake	Median	p25, p75	Median	p25, p75	Median	p25, p75	*P*
Energy (kJ)	7719	6276, 9506	7719	6257, 9552	7715	6330, 9452	0·99
Protein (E%)	16	15, 18	16	15, 18	16	14, 18	0·10
Carbohydrate (E%)	48	45, 51	48	45, 52	48	45, 51	0·73
Fibre (g/MJ)	2·7	2·1, 3·3	2·8	2·2, 3·4	2·7	2·0, 3·2	0·007
Fat (E%)	33	30, 36	33	30, 36	33	30, 36	0·34
Monounsaturated fat (E%)	12	10, 13	11	10, 13	12	10, 13	0·16
Polyunsaturated fat (E%)	5	4, 6	5	4, 6	5	4, 6	0·07
Saturated fat (E%)	13	12, 15	13	12, 15	14	12, 15	0·14
*n*-3 fatty acids (E%)	0·12	0·06, 0·02	0·11	0·06, 0·19	0·13	0·07, 0·21	0·05
EPA (g)	0·06	0·04, 0·12	0·06	0·04, 0·12	0·06	0·04, 0·11	0·56
DHA (g)	0·19	0·12, 0·34	0·19	0·12, 0·35	0·20	0·12, 0·33	0·66
Vitamin A (RE)	737	608, 901	738	614, 906	729	604, 894	0·37
Vitamin D (µg)	5·7	4·5, 7·0	5·6	4·6, 7·1	5·8	4·5, 7·0	0·54
Vitamin E (mg)	9·1	7·8, 10·8	9·1	7·9, 10·9	9·0	7·8, 10·6	0·27
Thiamine (mg)	1·4	1·2, 1·7	1·4	1·2, 1·7	1·3	1·2, 1·6	0·013
Riboflavin (mg)	2·0	1·7, 2·3	2·0	1·7, 2·3	2·0	1·7, 2·2	0·16
Niacin (NE)	31·5	28·4, 34·7	31·7	28·5, 34·8	31·4	28·3, 34·5	0·43
Vitamin B_6_ (mg)	2·0	1·7, 2·3	2·1	1·8, 2·3	2·0	1·7, 2·3	0·14
Vitamin B_12_ (µg)	5·0	4·1, 6·0	5·0	4·1, 6·1	5·0	4·0, 6·0	0·53
Vitamin C (mg)	122	89, 161	125	92, 162	117	86, 157	0·13
Phosphorus (mg)	1468	1320, 1608	1482	1334, 1620	1440	1293, 1586	0·007
Folate (µg)	324	276, 381	327	280, 384	316	264, 369	0·02
Fe (mg)	11·8	9·9, 13·7	12·0	10·1, 13·8	11·5	9·6, 13·3	0·009
Ca (mg)	1052	905, 1248	1063	919, 1260	1044	893, 1211	0·05
Potassium (mg)	3232	2887, 3574	3260	2903, 3594	3196	2862, 3555	0·13
Mg (mg)	355	312, 401	361	316, 411	346	303, 387	<0·001
Se (µg)	41·4	35·3, 47·1	41·5	35·7, 47·6	41·1	34·2, 46·9	0·37
Zn (mg)	10·6	9·6, 11·5	10·7	9·7, 11·6	10·4	9·4, 11·4	0·01

any-SU, any supplement users; non-SU, non-supplement users; E%, energy percentage.

* Assessed by Mann Whitney U test.

For dietary intake (i.e. from diet alone) among all women, adherence to AR was high (60–100 %) for most nutrients, except for vitamin D where only 24 % reached AR (Table 4). Adherence to RI among all women was lowest for dietary intake of vitamin D (6 %), folate (10 %), Fe (23 %) and Se (10 %). None of the women met recommendation of ≥ 1 E% of *n*-3 fatty acids per day from diet alone. Dietary intakes below LI were reported by 8 % for vitamin A, 5 % for vitamin D, 1 % for riboflavin, 7 % for B_12_ and 2 % for Fe and Se. All women reported dietary intakes below UL, except 1 % for Ca. Few differences in proportions of adherence to AR and RI for dietary intake between any supplement users and non-users were found (Table 4). A higher proportion of adherence to AR for dietary intake of Zn, and to RI for dietary intake of fibre, polyunsaturated fat, thiamine and Mg were found among supplement users compared with non-users.

**Table 4.** Percentage of women adhering to recommended intake and average requirement among all women, any supplement users and non-supplements users

									Differences* in proportion between any-SU and non-SU	
			All women (*n* 1044)		any-SU (*n* 665)		non-SU (*n* 379)		≥ or < RI	≥ or < AR
Nutrient	RI†	AR‡	≥ RI %	≥ AR %	≥ RI %	≥ AR %	≥ RI %	≥ AR %	*P*	*P*
Protein (E%)	10–20		93		93		93		0·77	
Carbohydrate (E%)	45–60		71		71		72		0·71	
Fibre (g/MJ)	> 3		38		41		34		0·02	
Fat (E%)	30–45		86		87		85		0·23	
Monounsaturated fat (E%)	10–25		79		78		81		0·28	
Polyunsaturated fat (E%)	5–10		45		47		41		0·05	
Saturated fat (E%)	< 10		10		10		9		0·55	
*n*-3 fatty acids (E%)	≥ 1		0		0		0			
DHA (g)	0·2		48		48		49		0·78	
Vitamin A (RE)	800	500	39	82	39	83	38	81	0·72	0·52
Vitamin D (µg)	10	7·5	6	21	6	20	6	23	0·87	0·33
Vitamin E (mg)	10	5	37	94	38	94	35	94	0·26	0·95
Thiamine (mg)	1·5	0·9	39	88	42	88	35	86	0·04	0·39
Riboflavin (mg)	1·6	1·1	70	95	71	96	68	93	0·29	0·11
Niacin (NE)	17	12	98	100	98	100	98	100	0·60	–
Vitamin B_6_ (mg)	1·5	1	79	98	80	98	77	97	0·31	0·37
Vitamin B_12_ (µg)	2	1·4	98	99	98	99	98	99	0·88	0·53
Vitamin C (mg)	85	50	74	93	75	94	73	91	0·45	0·05
Phosphorus (mg)	700	450	99	100	99	100	98	100	0·06	–
Folate (µg)	500	200	10	89	11	90	10	87	0·59	0·20
Fe (mg)	15§	10	23	60	24	61	21	59	0·20	0·40
Ca (mg)	900	500	63	97	65	97	61	97	0·20	0·85
Potassium (mg)	3·1		51		52		49		0·38	
Mg (mg)	280		73		75		69		0·05	
Se (µg)	60	30	10	79	10	79	10	78	0·92	0·67
Zn (mg)	9	5	65	99	66	100	62	99	0·24	0·02

any-SU, any supplement users; non-SU, non-supplement users; RI, recommended intake; AR, average requirement; E%, energy percentage.

* Assessed by *χ* ^2^ test.

† RI for pregnant women in the Nordic Nutrition Recommendations 2012^(32)^.

‡ AR for women in the Nordic Nutrition Recommendations 2012^(32)^.

§ RI for women in reproductive age in the Nordic Nutrition Recommendations 2012. Pregnancy requires 500 mg of stored Fe^(32)^.

**Fig. 2.**
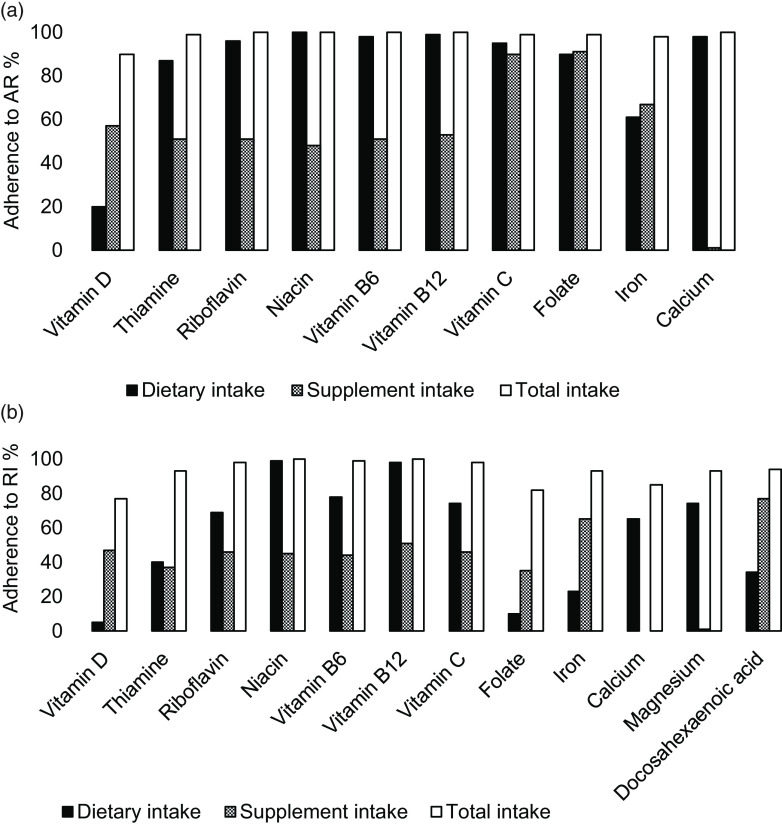
Among nutrient-specific supplement users, percentage of women adhering to average requirement (AR) (a) and recommended intake (RI) (b) from dietary, supplement and total intake.

When estimating total intake (both dietary- and nutrient-specific supplement intake), supplement users reached AR for all nutrients (Fig. 2(a)), except for vitamin D, where 15 % were below AR. The proportions with total intakes above RI ranged between 75 and 100 %, except for *n*-3 fatty acids, where only 2 % met RI (Fig. 2(b)). No one reported total intakes below LI, and UL was only exceeded for Fe by 30 %. Estimated median intakes of specific nutrients from supplements are found in online Appendix Table S4.

**Fig. 3.**
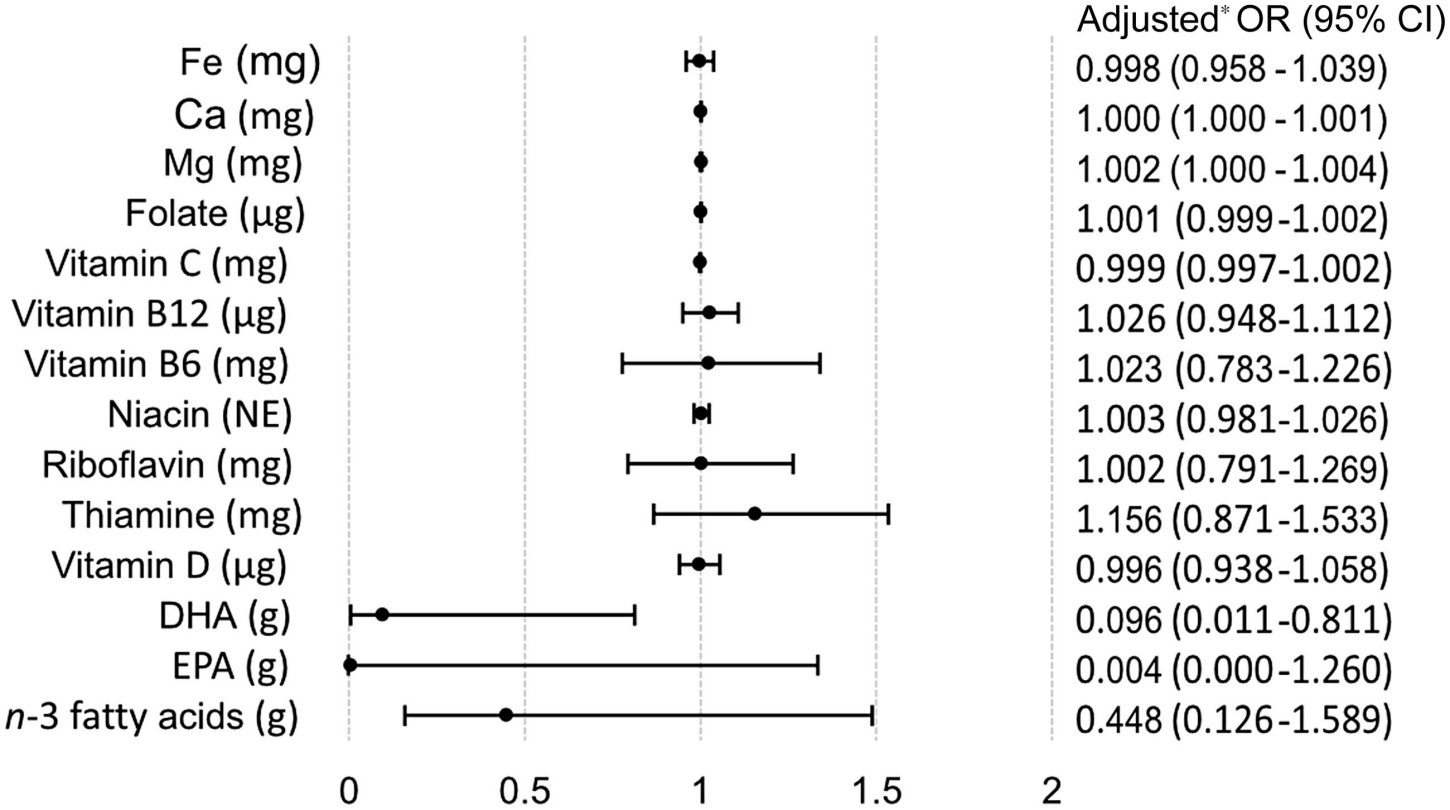
Multivariable logistic regression analyses of estimated dietary intake and the probability of nutrient-specific supplement use compared with non-supplement use. * Adjusted for total energy intake, age, BMI, gestational weight gain, education, parity and origin.

The logistic regression analyses of dietary intake of specific nutrients and supplement use of these nutrients showed that women with higher energy adjusted dietary intake of Mg were more likely to report use of Mg supplements (OR = 1·002, 95 % CI 1·000, 1·004, *P* = 0·026) (Fig. 3). Conversely, women with higher energy adjusted intake of DHA were less likely to report use of *n*-3 fatty acids supplements (OR = 0·096, 95 % CI 0·011, 0·811, *P* = 0·031). The crude logistic regression models of energy-adjusted dietary intake and supplement use of specific nutrients are found in online Appendix Fig. S1.

## Discussion

This study found that a majority of pregnant women reported use of dietary supplements in third trimester, of which Fe, folic acid and vitamin D were the most commonly reported. Adherence to AR from dietary intakes alone was high for most nutrients, whereas less than one-fourth of the women adhered to RI of vitamin D, folate, Fe and Se. Women who used any type of supplement reported a higher energy-adjusted dietary intakes of thiamine, phosphorus, folate, Fe, Mg and Zn, compared with non-users. Among non-users, a minority reached RI of vitamin D, folate and Fe from their diet. In contrast, a majority of supplement users had total intakes above RI for most nutrients.

Multivitamin-mineral supplements are not generally recommended during pregnancy in Sweden, but was reported by 40 % of the participants, which is similar with earlier findings in pregnant women in Sweden^(24,25)^. We found that supplemental vitamin D and folic acid were taken mostly as multivitamin-mineral formulations, and the contribution from supplements to meet RI for these nutrients was substantial. However, for vitamin B complex and vitamin C, a large proportion of supplement users were already meeting RI from diet alone. Thus, use of multivitamin-mineral supplements including these nutrients may have resulted in unnecessary supplement use, suggesting that individualised guidance for dietary counselling and supplementation guidance during pregnancy is needed. Recently, a study from the USA provided estimates for supplement doses of key nutrients for pregnant women including vitamin A and D, folate, Fe, Ca and *n*-3 fatty acids to complement dietary intake^(37)^. The study indicated a lack of available prenatal supplements that contain nutrients in appropriate doses in relation to estimated nutrient requirements during pregnancy. Similar studies conducted in Sweden are lacking.

Previous studies have found supplement to be associated with excessive intake of certain nutrients^(14,15,38–40)^. For example, excessive intake of vitamin A during pregnancy is of special concern due to its teratogenic effect and, thus, an increased risk of fetal malformations^(41)^. Furthermore, excessive intake of vitamin D during pregnancy is linked to the risk of hypercalcaemia^(42)^, while excessive intake of iodine might have negative effects on thyroid function^(43)^. However, in the current study, none of the reported total intakes exceeded UL, except for Fe. This result is not surprising since the UL of 60 mg Fe per day is set for non-pregnant women, because UL for pregnant women is lacking^(32)^. Routine screening for Fe deficiency is a standard practice within Swedish antenatal care^(12)^. Hence, high dose of Fe supplements likely indicates treatment of identified Fe deficiency. Due to the lack of pregnancy-specific Fe recommendation in Nordic Nutrition Recommendations 2012, we applied the recommendation for women in reproductive age^(28)^. It is worth noting that this recommendation does not fully address the Fe needs during pregnancy. If a higher RI had been used, such as the recommended daily allowance of 27 mg Fe in the USA^(44)^, a larger proportion of the participants would not have met the RI compared with the results presented here (23 %).

Use of vitamin D supplements was reported by 45 % of the participants. Among women using vitamin D supplements, 15 % still reported a total vitamin D intake below AR and 23 % below RI. This is explained by the combination of low dietary intake of vitamin D and the low doses of vitamin D in many multivitamin-mineral supplements. The challenge of reaching RI from dietary sources alone in the Swedish population has been reported earlier, both in pregnancy^(24,45)^ and in the general adult population^(29)^. Since these studies were conducted, an expansion of the mandatory fortification programme was implemented in Sweden to increase vitamin D intake at a national level^(46)^. So far, the effects of the expanded fortification programme have not been evaluated. Nevertheless, our results suggest that dietary intake of vitamin D is low, and that pregnancy-specific recommendations of vitamin D supplementation, similar to recommendations in Denmark and Finland^(47)^, may be justifiable.

We found that nearly 90 % of all women adhered to AR of folate from reported dietary intake alone. Noticeably, only 10 % adhered to RI of 500 µg per day during pregnancy. The low dietary intake of folate is consistent with results from previous Swedish studies, reported both in early^(25)^ and late pregnancy^(24)^. However, when accounting for total folate intake from both diet and supplements, 80 % of folic acid supplement users reached RI. Thus, dietary intakes alone are insufficient and supplementation with folic acid might be warranted to reach RI^(24,25)^.

None of the women in the current study reported dietary intakes above RI for *n*-3 fatty acids and less than 50 % reported dietary intakes above RI for DHA. The low adherence to RI of *n*-3 fatty acids could possibly be explained by a decreased consumption of fish due to concerns about methyl mercury and dioxin intakes among pregnant women. A negative association between dietary intake of DHA and the use of *n*-3 fatty acid-specific supplements was found, indicating that women with a higher reported dietary intake of DHA were less likely to use *n*-3 fatty acid supplements. However, this result should be interpreted with some caution due to the low prevalence of reported *n*-3 fatty acid supplement use (5 %).

One of the strengths of the current study is the relatively large sample size of the included women in the analysis. The GraviD study is a population-based cohort, but since the web-based FFQ was only available in Swedish, there is a risk of selection bias among the included women in the current analyses. However, the characteristics of the included women were similar to the overall GraviD cohort in terms of age, BMI in first trimester, GWG, nulliparity and tobacco use^(48)^.

Limitations of the current work are mainly related to the nature of self-reported data. For both dietary intake and supplement use, both under- and overreporting may exist^(49)^. Results from the validation studies^(26,27)^ showed that the FFQ underestimate intake of energy and most nutrients compared with 7-d weighed food record. Taking this into account, nutrient intake was in our analyses calculated as relative intakes, that is, energy percentage and median intakes adjusted for total energy intake. The ability of the FFQ to accurately capture adherence to AR and RI is unclear. A further aspect is that the FFQ has only been validated among non-pregnant women^(26,27)^, with the exception for vitamin D intake^(50)^. In addition, the questions on the supplements have not been validated; however, correlations between self-reported supplement use and biomarkers have previously been found in pregnancy^(51)^. Furthermore, estimated doses and brands of supplements were applied for some of the supplement users where such data were missing. The imputation may potentially have led to inaccurate estimations of supplement intake, particularly concerning multivitamin-mineral supplements, which has previously been found to vary in nutrient content between different brands^(37)^. However, the missing brands of multivitamin-mineral supplements were rather low, and therefore, they are not considered to invalidate the conclusions. Overall, reported estimates of dietary intake and intake of specific nutrients from supplements should therefore be interpreted with some caution. The FFQ was administered in gestational week > 31 and captured dietary intake over the preceding 2 months. Consequently, conclusions regarding nutrient intake during pregnancy can only be drawn for this specific period and not for the first half of pregnancy. Lastly, a high proportion of the participants was highly educated, which might decrease the external validity of the results. We suggest that future studies on dietary and supplemental intake during pregnancy should focus on identifying women who are particularly at high risk of inadequate nutritional intake and status, and thereafter targeted interventions could be developed to address their specific needs.

In conclusion, we found that adherence to AR from diet alone was high for most nutrients among pregnant women in the south-west of Sweden, whereas adherence to RI of vitamin D, folate, Fe and Se was low. Supplement use contributed substantially to reaching RI for vitamin D, folate and Fe, highlighting that diet alone may be inadequate to reach RI for these nutrients. Supplement users had a higher dietary intake for several nutrients than non-users had. There is a need for increased awareness of adequate dietary intake during pregnancy and more individualised guidance on supplement use.

## Supplementary material

For supplementary material accompanying this paper visit https://doi.org/10.1017/S0007114523001794. click here to view supplementary material
