# Supplementary material for: Supplement use in relation to dietary intake in pregnancy: an analysis of the Swedish GraviD cohort
Source: Br J Nutr. 2023 Aug 11;131(2):256–64. doi: 10.1017/S0007114523001794 (PMC10751946; doi:10.1017/S0007114523001794)
Supplement: Supplementary file 1 [file S0007114523001794sup001.docx]

**Online Supplementary Material**

**Supplement use in relation to dietary intake in pregnancy – an analysis of the Swedish GraviD cohort**

Mathilda Forsby^1^, Anna Winkvist^1^, Linnea Bärebring^1^, Hanna Augustin^1^

^1^Institute of Medicine, University of Gothenburg, Gothenburg, Sweden.

**Corresponding author**

Mathilda Forsby; mathilda.forsby@gu.se

**Supplementary table S1.** Coding of supplements.

| **Any supplement** | **Single nutrient supplement** | **Single nutrient supplement and/or part of multivitamin-mineral supplement** | **Other** |
| --- | --- | --- | --- |
| One or more regardless of dose and frequency | N-3 fatty acids | Vitamin D, calcium and vitamin D, multivitamin-mineral containing vitamin D | Herbs, aloe vera, nettles, algaes |
|  |  | Folic acid, multivitamin-mineral containing folic acid |  |
|  |  | Vitamin B complex, B6, B12 (not folic acid), multivitamin-mineral containing vitamin B |  |
|  |  | Vitamin C, multivitamin-mineral containing vitamin C |  |
|  |  | Magnesium, calcium and magnesium, multivitamin-mineral containing |  |
|  |  | Calcium, calcium and vitamin D, calcium and magnesium, multivitamin-mineral containing calcium |  |
|  |  | Iron, multivitamin-mineral containing iron |  |

**Supplementary table S2.** Reported use of single supplements and the estimations that were made when corresponding brand or dose were missing.

| **Reported supplement** | **Reported use of a single nutrient supplement**  **% (n)** | **Estimated content of supplement** | **Proportion of supplements with estimated content % (n)** |
| --- | --- | --- | --- |
| Multivitamin-mineral | 40 (412) | When a corresponding brand was missing, the brand of the most common reported prenatal multivitamin-mineral was used. | 27 (113) |
| Vitamin D | 6 (62) | When frequency was recorded but not dose, an estimated dose of 10 µg was used. The rationality was that this was the most common dose of reported single vitamin D supplements. | 45 (28) |
| Vitamin C | 1 (8) | When frequency was recorded but not dose, an estimated dose of 1000 mg was used. The rationality was that this was the most common dose of single vitamin C supplements. | 88 (7) |
| Folic acid | 11 (117) | When frequency was recorded but not dose, an estimated dose of 400 µg was used. The rationality was that 400 µg is the recommended dose of folic acid during pregnancy in Sweden. | 74 (86) |
| Vitamin B complex (vitamin B12) | 2 (16) | When frequency was recorded but not dose, an estimated dose of 1000 µg was used. The rationality was that 1000 µg was the most common dose of single vitamin B12 supplements. | 38 (6) |
| Magnesium | 1 (10) | When frequency was recorded but not dose, an estimated dose of 125 mg was used. The rationality was that 125 mg was the most common dose of single and multivitamin-mineral magnesium supplements. | 70 (7) |
| Calcium | 1 (6) | When frequency was recorded but not dose, an estimated dose of 500 mg was used. The rationality was that 500 mg was the most common dose of single calcium supplements. | 50 (3) |
| Iron | 21 (216) | When frequency was recorded but not dose, an estimated dose of 100 mg was used. The rationality was that 100 mg was the most common dose of single iron supplements. | 10 (22) |
| N-3 fatty acids | 5 (53) | When a corresponding brand was missing, an estimated brand of the most common reported n-3 fatty acid supplements was used. | 72 (38) |

**Supplementary table S3.** Nutrient content per recommended dose per day (two pills) in the most commonly used prenatal multivitamin-mineral supplement among supplement users.

| **Nutrient** | **Content** |
| --- | --- |
| Vitamin A (RE) | 800 |
| Vitamin D (µg) | 10 |
| Vitamin E (mg) | 30 |
| Vitamin K (µg) | 90 |
| Thiamine (mg) | 1.6 |
| Riboflavin (mg) | 1.7 |
| Niacin (NE) | 20 |
| Folic acid (µg) | 500 |
| Vitamin B6 (mg) | 1.6 |
| Vitamin B7 (µg) | 30 |
| Vitamin B12 (µg) | 2.6 |
| Pantothenic acid (mg) | 5 |
| Vitamin C (mg) | 140 |
| Iron (mg) | 15 |
| Calcium (mg) | 300 |
| Magnesium (mg) | 120 |
| Selenium (µg) | 55 |
| Zinc (mg) | 9 |
| Iodine (µg) | 175 |
| Copper (mg) | 1 |
| Manganese (mg) | 2 |
| Chromium (μg) | 30 |
| Molybdenum (μg) | 45 |

**Supplementary table S4.** Estimated micronutrient intake from supplements among nutrient specific supplement users.

|  | **% (n)** | **Median (p25, p75)** |
| --- | --- | --- |
| Vitamin D (µg) | 45 (469) | 7.5 (5, 10) |
| Thiamine (mg) | 42 (434) | 1.1 (0.8, 1.6) |
| Riboflavin (mg) | 42 (434) | 1.3 (0.85, 1.7) |
| Niacin (NE) | 42 (434) | 10 (9, 20) |
| Vitamin B6 (mg) | 42 (434) | 1.2 (0.8, 1.6) |
| Vitamin B12 (µg) | 42 (434) | 2 (1.3, 2.6) |
| Vitamin C (mg) | 40 (420) | 70 (65, 140) |
| Folic acid (µg) | 50 (518) | 400 (250, 500) |
| Iron (mg) | 52 (547) | 15 (7.5, 65) |
| Calcium (mg) | 38 (400) | 150 (150, 300) |
| Magnesium (mg) | 39 (403) | 60 (60, 120) |
| N-3 fatty acids (g) | 5 (53) | 0.66 (0.66-0.66) |
| Docosahexaenoic acid (g) | 5 (53) | 0.32 (0.18-0.32) |
| Eicosapentaenoic acid (g) | 5 (53) | 0.24 (0.22-0.24) |


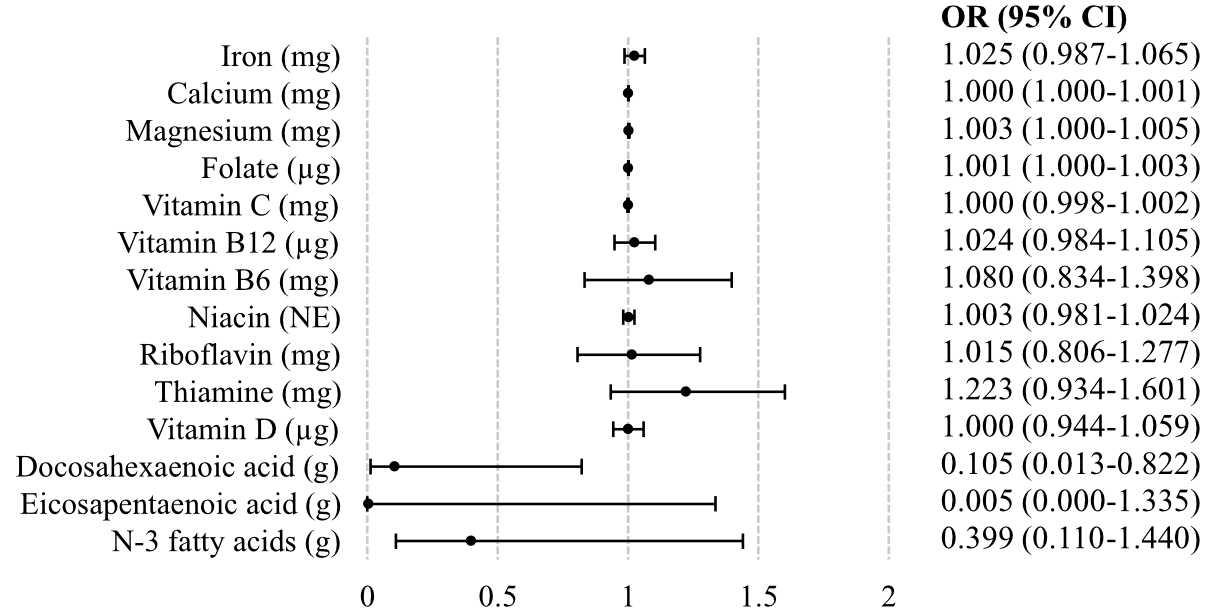


**Supplementary figure S1.** Univariable logistic regression analyses of estimated dietary intake adjusted for total energy intake and the probability of nutrient specific supplement use compared to non-supplement use.

Abbreviations: OR, odds ratio; CI, confidence interval.
